# Grasping Force Control of Multi-Fingered Robotic Hands through Tactile Sensing for Object Stabilization

**DOI:** 10.3390/s20041050

**Published:** 2020-02-14

**Authors:** Zhen Deng, Yannick Jonetzko, Liwei Zhang, Jianwei Zhang

**Affiliations:** 1 School of Mechanical Engineering and Automation, Fuzhou University, Fuzhou 350108, China; yuchen8807@163.com; 2 TAMS, Department of Informatics, University of Hamburg, D-22527 Hamburg, Germany; jonetzko@informatik.uni-hamburg.de (Y.J.); zhang@informatik.uni-hamburg.de (J.Z.)

**Keywords:** grasping force control, force estimation, slip detection, multi-fingered robotic hands

## Abstract

Grasping force control is important for multi-fingered robotic hands to stabilize the grasped object. Humans are able to adjust their grasping force and react quickly to instabilities through tactile sensing. However, grasping force control through tactile sensing with robotic hands is still relatively unexplored. In this paper, we make use of tactile sensing for multi-fingered robot hands to adjust the grasping force to stabilize unknown objects without prior knowledge of their shape or physical properties. In particular, an online detection module based on Deep Neural Network (DNN) is designed to detect contact events and object material simultaneously from tactile data. In addition, a force estimation method based on Gaussian Mixture Model (GMM) is proposed to compute the contact information (i.e., contact force and contact location) from tactile data. According to the results of tactile sensing, an object stabilization controller is then employed for a robotic hand to adjust the contact configuration for object stabilization. The spatio-temporal property of tactile data is exploited during tactile sensing. Finally, the effectiveness of the proposed framework is evaluated in a real-world experiment with a five-fingered Shadow Dexterous Hand equipped with BioTac sensors.

## 1. Introduction

Stable grasping of unknown objects is one of the fundamental abilities for robots performing manipulation tasks in daily-life environments. External disturbances caused by environmental changes may occur when a robotic hand grasps objects. This may cause a planned-to-be stable grasp into an unstable one. Maintaining the stability of grasped objects over time is critically essential for further robotic manipulations. Humans are capable of reacting to instabilities quickly through tactile sensing by adjusting their grasp configurations and therefore improve stability. Studies from neuroscience have demonstrated that tactile perception provides critical information about the object’s physical properties and the contact event between the object and the hand [1]. However, grasping force control through tactile sensing for robotic hands is still relatively unexplored. To stabilize a grasped object effectively, three related problems should be addressed jointly: (1) How to detect properties of the grasped object (i.e., material); (2) How to perceive contact events in real-time (i.e., slippage); (3) How to adjust the grasping force of a robotic hand through tactile sensing to avoid slippage or to improve grasp stability. In this work, we combine a tactile sensing method with a grasping force controller to address the three important problems above.

The goal of grasping force control is to maintain contact between the robotic hand and the grasped object to avoid slippage. A slip occurs when the grasping force applied by the robotic hand is insufficient. It may be caused by the slipperiness of the object or an external disturbance. Hence, online slip detection is of critical importance for robots to stabilize grasped objects. Once slippage is detected, the robotic hand needs to adjust its contact configuration. Tactile information has been used as the primary sensory modality for slip detection [2]. Most of the previous works formulated slip detection as a classification problem, in which a classifier was built with an SVM [3] or random forest [4]. Those works typically require designing features manually from tactile data for classification. To achieve a better detection performance, more recent works applied deep learning techniques for slip detection [5,6,7]. In those works, features were automatically learned from tactile data for classification. In addition, recognizing the object’s physical properties (e.g., material) is also critical for object stabilization. Tiest et al. [8] demonstrated that the main mechanism for modulation of grip force in the early phase of grasping is the real-time sensation of the material (texture) of an object. Information about object material could help robotic hands to know how to interact with them in advance. Although there has been some previous work [9,10,11] that implemented material detection, few works have incorporated material detection into the grasping force controller. In general, there are two main differences compared with the state-of-the-art methods: (1) In contrast to previous methods that consider slip and material detection separately. This work trains an online detection module to identify contact events and material simultaneously. (2) An online detection module is designed based on a deep neural network for slip and material detection. It is not necessary to design features manually for slip or material detection.

Grasping force control of robtoic hands in this work is implemented through a force feedback controller. This controller drives the robotic hand to adjust its grasping force so as to track the desired contact configuration and to avoid slippage [12,13]. One crucial technique of the force feedback control is to estimate the contact force from tactile data, which is taken as a feedback signal for the feedback controller [14,15]. Owing to the high-dimensionality of tactile data, most of the previous works define the contact force as a weighted sum of tactile data [14,15,16]. Those works ignore the computation of the contact region on the tactile sensor. As a result, the estimated force is easily affected by the non-contact tactile array or electrode. This work proposes determining the contact region on the sensor surface and then estimates the contact information (i.e., contact force and contact location) based on the electrodes belonging in the contact region. This work defines a tactile image according to the distributed electrodes of the tactile sensor. The tactile image describes the pressure distribution when the robotic hand makes contact with the sensor. By exploiting the spatial property of tactile data, the proposed method reduces the uncertainties caused by the non-contact electrodes. Most of the previous methods used a fixed grasping force predefined manually as the reference force for feedback control. Those methods were limited to handling similar or known objects and were unable to grasp unknown ones. To avoid this limitation, this work proposes integrating force feedback control with tactile sensing. The desired grasping force is updated automatically according to the result of the slip and material detection.

In this work, the objective is to enable a robotic hand to adjust its grasping force automatically according to the measurements of slip, object material, and contact force from tactile data. To this end, we propose making use of tactile sensing for a multi-fingered robotic hand to stabilize unknown objects stably without prior knowledge of their shape or physical properties. This work addresses the problems of slip detection, object material detection, and force feedback control jointly. We first detect the contact event (slippage or not) and object material. The contact force between the hand and the grasped object is also estimated from tactile readings produced by a BioTac sensor. The spatio-temporal property of tactile data is exploited for tactile sensing. The proposed sensing methods are developed and validated using data obtained from BioTac sensors. According to the result of tactile sensing, the force feedback controller is employed to drive the robotic hand to adjust its grasping force online. The performance of the proposed mehtods is demonstrated with a Shadow Dexterous Hand equipped with BioTac sensors. The following contributions are made in this work:

1. An online detection module is introduced, which trains a Deep Neural Network (DNN) to detect contact events and object materials simultaneously from tactile data. A tactile dataset is collected for slip and material detection. The dataset contains tactile data, contact event, and material information.
2. A force estimation method based on GMM is proposed to calculate the contact information (i.e., contact force and contact location) from tactile data. This method considers the computation of the contact region on the surface of the tactile sensor.
3. A grasping force controller is proposed for a multi-fingered robotic hand to stabilize the grasped object. It integrates tactile sensing with force feedback controller to adjust the grasping force between the robotic hand and the object online.

The rest of the article is organized as follows. Section 2 presents related work. Section 3 introduces the object stabilization framework and its three main components (i.e., online detection module, force estimation, and grasping force controller). Experiments and results are presented in Section 4. Finally, the conclusions and future work are introduced in Section 5.

## 2. Related Work

Slip detection from tactile information has been implemented in previous works. Most of the previous works formulated slip detection as a classification problem where relevant features were extracted from tactile data to train a classifier. Li et al. [17] used a GMM to build a classifier. Chebotar et al. [3] used a Spatio-Temporal Hierarchical Matching Pursuit (ST-HMP) to create a feature descriptor for tactile data and employed Support Vector Machine (SVM) to train a classifier. In addition, Veiga et al. [4] predicted the contact event by using Random Forest (RF). Those methods required hand-crafted features that are difficult to design manually. To achieve a better performance of slip detection, more recent works applied deep learning techniques for slip detection. Previous works [18,19] demonstrated that Long Short-Term Memory (LSTM) was suitable for processing time-series data. Van Wyk et al. [5] developed an LSTM-based neural network to learn a slip classifier which took tactile data as input and predicted slippage or not. In addition, Zapata-Impata et al. [6] proposed a ConvLSTM to learn spatio-temporal tactile features of tactile data for the direction of slip detection. Those works all took advantage of LSTM to extract the relevant features automatically from tactile data. Different from previous methods that consider a single slip detection task, this work proposes an online detection module that implements slip detection and material detection jointly.

Some research has used vision-based tactile sensors to implement slip detection. Those sensors typically used micro-cameras to capture the interaction between the robotic hand and objects. Yuan et al. [20] used a GelSight sensor to measure shear and slip on a contact surface. In that work, they analyzed the sequence of images of GelSights elastomer medium and detected the conditions of contact based on the deformation under the external load. Donlon et al. [21] extended the GelSight sensing technique and developed a tactile-vision finger. Some works used machine learning methods to analyze images captured by a tactile-based sensors. Zhang et al. [22] used a optical-based tactile sensor, i.e, FingerVision, to capture images and trained a convolution Long Short Term Memory (convolutional LSTM) networks for slip detection. Vision-based tactile sensors can capture the image of the object surface directly. Compared with Vision-based tactile sensors, the advantage of the BioTac sensor is that the BioTac sensor is very sensitive to contact with objects and the sampling rate of the BioTac sensor can be 1000 Hz. Therefore, the BioTac sensor is suitable for force estimation and slip detection. Both of those tasks (i.e., force estimation and slip detection) require sensors to perceive changes in object state or robot state quickly. In this work, we make use of the BioTac sensor for tactile sensing.

Material detection from tactile information is essential for robots, which identify object properties, such as texture, hardness, or friction [23]. Chu et al. [9] proposed Hidden Markov Models (HMM) to analyze the temporal fluctuation of tactile data and trained a linear SVM to detect object material. In addition, Liu et al. [24] used a sparse kernel coding algorithm to compute the relevant features of tactile data for object recognition. Schneider et al. [25] applied a bag-of-words approach to process tactile data for object identification. Recently, Convolutional Neural Networks (CNN) have achieved outstanding performance in solving computer vision problems [26]. Han et al. [11] proposed a multi-label detection model that took a tactile sequence as input and predicted object’s hardness, thermal conductivity, roughness, and texture simultaneously. There have also been some works that fused tactile and visual data for material detection. Gao et al. [10] proposed a DNN to fuse tactile and visual data to classify surfaces with haptic adjectives. In that work, the authors formulated material detection as a binary classification problem instead of multi-label classification problem. Moreover, those works do not encode the temporal property of tactile data. In this work, we employ an LSTM-based encoder–decoder to learn a representation of the tactile information for material detection, which captures the temporal property of tactile data and is low dimensional.

Force estimation from tactile information is a critical issue of object stabilization. The contact information (e.g., contact force and location) between the hand and the interacted object should be estimated from tactile data. Some methods inspired by the human sense of touch mechanism have been previously introduced. Romanon et al. [16] introduced a human-inspired grasp control method. In that work, robotic tactile signals were generated from pressure arrays to mimics human SA-I, SA-II, FA-I, and FA-II mechanoreceptors. The generated signals then guided the robotic hand to control the grasp force. Some other works define the contact force as the weighted sum of tactile measurements. Delgado et al. [15] proposed creating a tactile image using a mixture of Gaussian with dynamic deviations. The tactile image was used to compute the position and magnitude of the contact force, which guided the grasp adaptation. There have been some other works that learned a function mapping from tactile data to contact forces. Su et al. [14] learn the mapping function with a single-hidden-layer neural network. However, those learning-based methods required a considerable amount of training data for model training. Moreover, the trained models were unable to generalize to new sensors. The work at hand is inspired by the work from Delgado et al. [15] and Zapata-Impala et al. [6] that represent tactile data as tactile images. Different from these two works that use a weighted sum method, we first determine the contact region of the fingertip by using a Gaussian mixture model and calculate the force based on the electronic impedance with a high likelihood. In this way, the tactile data are more suitable for force feedback control.

Object Stablization with robotic hands has been studied under the literature of grasp adaptation and grasp optimization [4,27]. There have been several works that use tactile information to adjust the contact configuration of robotic hands to improve grasp stability. Traditional analytical-based methods mainly use tactile information to calculate grasp quality metrics, like form- or force-closure [28,29]. The quality metrics guide the robotic hands to adjust the grasp configuration [30]. These methods are often impractical in real-world grasping applications since they rely on accuracy object models that are difficult to obtain. To avoid this limitation, some other works proposed learning stabilization strategies directly from human demonstrations [17,31,32]. Dang et al. [31,32] built a grasping dataset that stored the stable grasp configurations and the corresponding local geometry of objects. In that work, grasp configurations were adjusted according to the measured similarity of object’s local geometry. In addition, Li et al. [17] implemented grasp adaptation based on an object-level impedance controller. The feasible impedance parameters were searched from an existing dataset. These methods based on similarity measure only work for similar or known objects. It was unable to handle unknown objects. More recent works exploited deep learning techniques to predict the grasp quality [33] or the contact event [6]. The grasp configurations of robotic hands were updated according to the predicted results to improve grasp quality or avoid slippage. Hogan et al. [33] used a DNN to learn a tactile-based grasp quality metric. The grasp configuration of the robotic hand was adjusted through local transformations to maximize the grasp quality. Most of these methods focused on the simple grasping task with a two-fingered robotic hand. In this work, we propose an object stabilization framework for a multi-fingered robotic hand. Moreover, the proposed framework combines tactile sensing techniques with object stabilization controller to ensure a stable grasp.

## 3. Methodology

**Figure 1:**
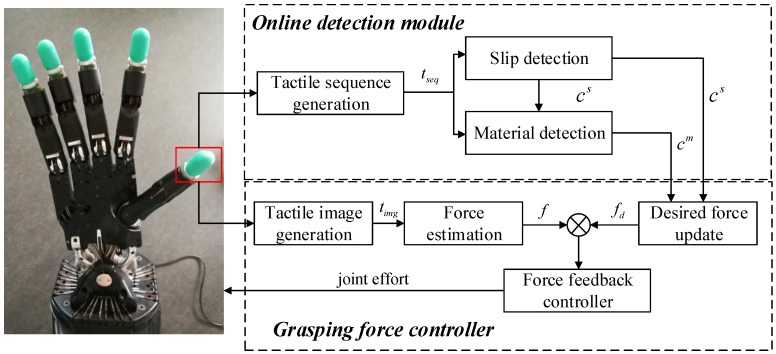
Overview of the proposed grasping force control framework. Firstly, the tactile sequence $t_{seq}$ and the tactile image $t_{img}$ are generated from tactile readings that produced by a tactile sensor. The contact event $c^{s}$ and the object material $c^{m}$ are detected with an online detection module. Next, the current contact force *f* is estimated from $t_{img}$. Finally, the desired grasping force $f_{d}$ is updated based on the detection results. The grasping force controller is employed to drive the robotic hand to track the $f_{d}$.

This section introduces the proposed grasping force control framework for a multi-fingered robotic hand to stabilize unknown objects. The proposed framework consists of three main components: an online detection module, a force estimation method, and an object stabilization controller. The detection module based on DNN samples tactile sequences online from tactile readings as inputs and predicts the object material and contact event simultaneously. A force estimation method based on GMM is employed to process the tactile data online and calculate the contact information (i.e., contact force and contact location). By exploiting the results of tactile sensing, a grasping force controller is employed to drive the robotic hand to adjust its contact configuration online to track the desired contact configuration or to avoid slippage. Figure 1 illustrates an overview of the proposed grasping force control framework.

### 3.1. Online Detection Module for Slip and Material Detection

This subsection presents an online detection module that takes a tactile sequence sampled from tactile readings as inputs and detects the contact event and the object material simultaneously. In the following, we first introduced the sampling process of the tactile sequence and then detailed the proposed online detection module.

**Figure 2:**
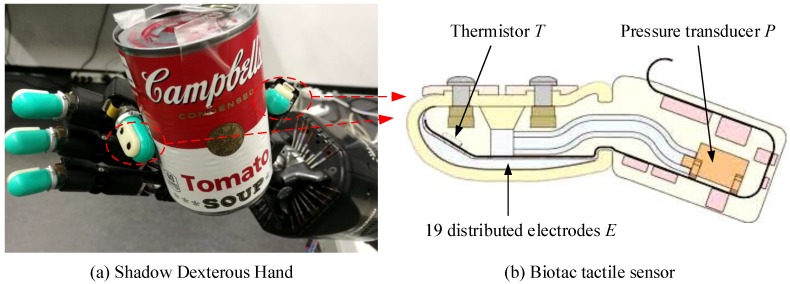
Shadow Dexterous Hand with BioTac sensors.

**Figure 3:**
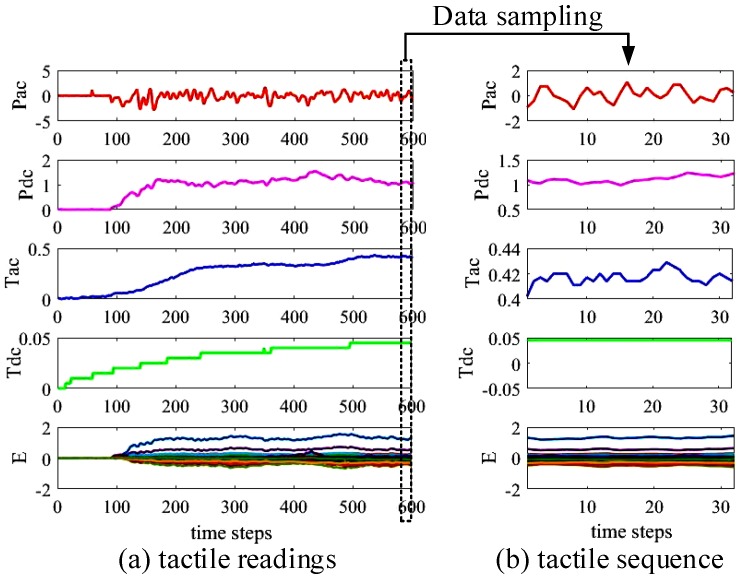
Example of a tactile sequence sampled from tactile readings. (**a**) tactile readings produced by the BioTac sensor; (**b**) tactile sequence with a dimension of 32∗23 sampled from tactile readings. The window size is set to 32 and the number of tactile features is 23.

In this work, we use a Shadow Dexterous Hand (https://www.shadowrobot.com/products/dexterous-hand/) equipped with BioTac sensors (https://www.syntouchinc.com/robotics/) for tactile data collection and experiments, as shown in Figure 2. The BioTac sensor is a multi-channel tactile sensor, which contains a bone like rigid core, surrounded by a flexible silicone skin filled with a conductive liquid. On the core of the BioTac sensor, three different sensor types are integrated: (1) a pressure transducer *P* for pressure sensing, (2) a thermistor *T* to measure temperature, (3) 19 distributed electrodes *E* which measure impedance and gather information about the deformation of the flexible hull. These multi-modal sensory capabilities provide 23-dimensional tactile features which includes absolute fluid pressure ($P_{dc}$), high-frequency fluid pressure ($P_{ac}$), core temperature ($T_{dc}$), core temperature change ($T_{ac}$), and 19 electrodes ($E_{1},\ldots ,E_{19}$). In the work at hand, tactile data are recorded from the BioTac sensor at 100 Hz. To obtain an input for slip and material detection, we sample a tactile sequence $t_{seq}$ with a window size *l* from the tactile readings, i.e., $t_{seq}=\left[P_{dc},P_{ac},T_{dc},T_{ac},E_{1},\ldots ,E_{19}\right]$. Thus, the tactile sequence $t_{seq}$ has a dimension of l∗23, where *l* is the window size and 23 is the number of tactile features. Figure 3 shows the sampling process of the tactile sequence $t_{seq}$. The tactile sequence $t_{seq}$ captures the temporal property of tactile data which is taken as the input of the proposed online detection module.

**Figure 4:**
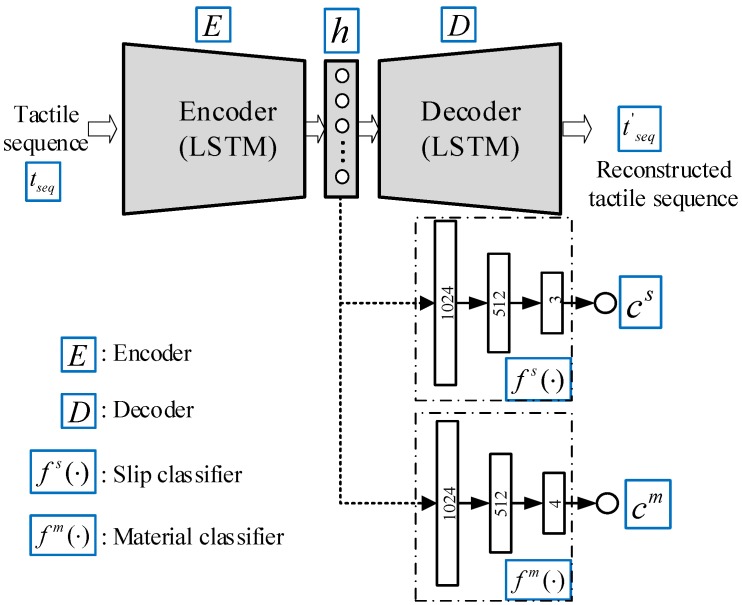
The architecture of the online detection module. The proposed detection module consists of three components: an LSTM-based encoder–decoder, a slip classifier, and a material classifier.

This work aims to solve a multi-task classification problem where the contact event and object material are detected simultaneously from tactile data. Instead of training two complex networks for the two detection tasks, this work proposed an online detection module. The architecture of the proposed detection module is illustrated in Figure 4. The proposed module first learns a low-dimension latent representation *h* of the tactile sequence and takes it as the input for slip and material classification. The use of the low-dimension latent representation *h* of the tactile sequence helps to reduce the model complexity and enables different classifiers to share the tactile features.

An LSTM-based encoder–decoder is firstly employed to learn the low-dimensional latent representation of a tactile sequence. The encoder–decoder consists of three parts: an encoder *E*, a latent representation *h*, and a decoder *D*. The encoder takes the tactile sequence $t_{seq}$ as input and outputs the latent representation *h* which is a feature vector with a fixed length. The decoder takes the latent representation *h* as input and reconstructs the inputted tactile sequence. Because the tactile sequence $t_{seq}$ is a time-series data, we take advantage of LSTM to capture the temporal characteristics of the tactile sequence $t_{seq}$. The LSTM is a recurrent neural network that excels at processing time-series data. Hence, this work employs LSTM recurrent units in the encoder and decoder. The LSTM unit takes the current frame $x_{t}$ and the previous hidden states $h_{t-1}$ as inputs and produces its hidden state $h_{t}$ and the output $o_{t}$. The forward pass of the LSTM unit is summarized as follows:

(1) $$\begin{array}{cc} i_{t} & =\sigma (W_{x,i}x_{t}+W_{hi}h_{t-1}+W_{c,i}c_{t-1}+b_{i})\\ f_{t} & =\sigma (W_{x,f}x_{t}+W_{h,f}h_{t-1}+W_{c,f}c_{t-1}+b_{f})\\ g_{t} & =tanh(W_{x,c}x_{t}+W_{h,c}h_{t-1}+b_{g}),\\ c_{t} & =f_{t}c_{t-1}+i_{t}g_{t}\\ o_{t} & =\sigma (W_{x,o}x_{t}+W_{h,o}h_{t-1}+W_{c,o}c_{t}+b_{o})\\ h_{t} & =o_{t}tanh\left(c_{t}\right) \end{array}$$

where σ and tanh are the sigmoid function and the hyperbolic tangent function, respectively. $c_{t}$ is the memory unit that stores the temporal information. The LSTM unit has four gates, i.e., the input gate $i_{t}$, the forget gate $f_{t}$, the cell gate $g_{t}$, and the output gate $o_{t}$. The four gates control the reading or modifying of the memory unit $c_{t}$. For more details of the LSTM unit, please refer to [18].

The training of the LSTM-based encoder–decoder is in an unsupervised setting. Hence, we only require tactile sequences $t_{seq}$ that are contained in the tactile dataset introduced in Section 4. In this work, the encoder–decoder runs through four tactile sequences ${\left\{t_{seq,i}\right\}}_{i=1:4}$ and produces four reconstructed tactile sequences ${\left\{t_{seq,i}^{{}^{\prime }}\right\}}_{i=1:4}$. The squared loss between the inputted tactile sequence and the reconstructed tactile sequence is calculated as the loss function for the training of the encoder–decoder, as defined in Equation (2):

(2) $$L_{1}=\sum_{i}^{4}\left|\right|t_{seq,i}-t_{seq,i}^{{}^{\prime }}{\left|\right|}_{2}^{2}$$

Next, we employ a slip classifier $f^{s}\left(\cdot \right)$ to achieve slip detection, which classifies the latent representation *h* of the tactile sequence produced by the encoder–decoder as one of the three different contact events $c^{s}$, as defined in Equation (3). In this work, the three different contact events are considered: *non-contact* $c_{non-c}^{s}$, *contact* $c_{contact}^{s}$, and *slip* $c_{slip}^{s}$. For slip classification, a feed-forward network is proposed to form the slip classifier $f^{s}\left(\cdot \right)$. Figure 4 shows the architecture of the proposed slip classifier. The network consists of three full-connected layers. We use a rectified linear unit (ReLU) as an activation function in the first two layers. The last full-connected layer is passed through a soft-max function to predict the class of the contact event. A cross-entropy function is used to define the loss function that is used to train the slip classifier $f^{s}\left(\cdot \right)$:

(3) $$c^{s}=f^{s}\left(h\right),\;c^{s}\in \left\{c_{non-c}^{s},c_{contact}^{s},c_{slip}^{s}\right\}$$

This work also trains a material classifier $f^{m}\left(\cdot \right)$ for object material detection, which classifies the latent representation *h* of the tactile sequence as one of four different materials $c^{m}$, as defined in Equation (4). The four different materials considered in this work are *paper* $c_{paper}^{m}$, *foam* $c_{foam}^{m}$, *plastic* $c_{plastic}^{m}$, and *metal* $c_{metal}^{m}$. The material classifier is trained with a feed-forward network which consists of three fully-connected layers. Figure 4 shows the architecture of the proposed material classifier. We use a rectified linear unit (ReLU) as an activation function in at the first two layers. The last fully-connected layer is passed through a soft-max function to predict the contact event class. A cross-entropy function is also used to define the loss function for training the material classifier:

(4) $$c^{m}=f^{m}\left(h\right),\;c^{m}\in \left\{c_{paper}^{m},c_{foam}^{m},c_{plastic}^{m},c_{metal}^{m}\right\}$$

The online detection module is trained with the tactile dataset proposed in this work. The training and evaluation process of the detection module is described in more detail in Section 4. The online detection module takes the tactile sequence $t_{seq}$ sampled from tactile readings as an input and outputs the detection results (i.e., contact event and object material) in real-time. These detection results are then used as guidance for the grasping force control.

### 3.2. Force Estimation from Tactile Information

**Figure 5:**
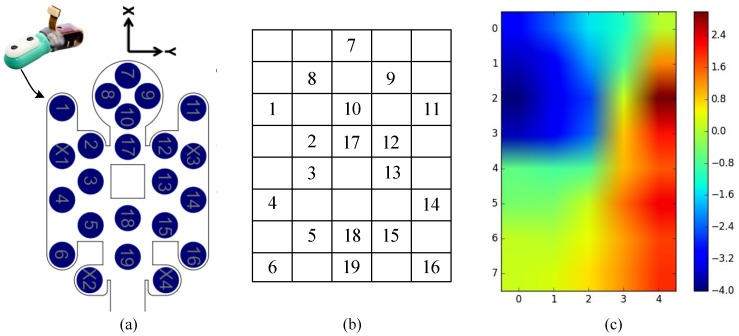
The definition of tactile image. (**a**) nineteen electrodes are distributed on the rigid core of the BioTac sensor; (**b**) the tactile image that consists of an 8∗5 matrix. The numbers denote the corresponding electrodes; (**c**) the tactile image after filling the empty pixels. The pixel value represents the electrode data.

One important technique for the grasping force control is the estimation of the contact force from tactile data. The estimated force is taken as a feedback signal for the grasping force control of the Shadow Hand. In this work, we use the BioTac sensors for tactile data collection and force estimation. This work proposes a force estimation method based on GMM that computes the contact information (i.e., contact force and contact location) of the robotic hand from the tactile data. In the previous subsection, the tactile sequence $t_{seq}$ that captures the temporal property of tactile data are sampled as the input of the proposed detection module. In terms of force estimation, we focus on the spatial property of tactile data. We take advantage of the electrodes data and their connectivity to compute the contact information. As introduced in Section 3.1, the BioTac sensor has nineteen electrodes distributed over its surface, as pictured in Figure 5a. To capture the spatial relationship among the nineteen electrodes, we define a tactile image $t_{img}$ that consists of an 8∗5 matrix in the *x*–*y* plane according to the locations of the nineteen electrodes, as shown in Figure 5b. The electrodes are arranged in a matrix-like distribution. In this work, the pixels of $t_{img}$ are filled as follows: when a pixel in $t_{img}$ corresponds to an electrode, we take the electrode data as the pixel value. Otherwise, the pixel value is filled with the mean of its surrounding non-empty values. Figure 5c shows an example tactile image $t_{img}$ after filling the empty pixels.

**Figure 6:**
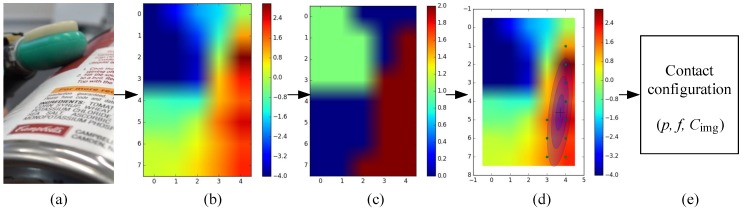
Estimation process of the contact information. (**a**) the BioTac sensor contacts with the object to produce tactile data; (**b**) the inputted tactile image $t_{img}$; (**c**) the tactile image is clustered based on GMM to obtain three different clusters denoted by different colors; (**d**) the contact region $C_{img}$ is segmented and a Gaussian distribution is used to fit the contact pixels; (**e**) the contact information between the fingertip and the object is calculated.

Next, the tactile image $t_{img}$ is used to calculate the contact information (i.e., contact force and contact location) between the fingertip and the object. From the tactile image shown in Figure 5c, it can be seen that there is only one region with a relatively high electrode value. This implies that the contact region is a part of the sensor surface, not the entire surface during physical interactions between the robotic hand and the object. Previous works mainly assumed that all the electrodes were supposed to contact the grasped object. In these works, the electronic values in the non-contact region were considered in the computation of the contact force. These non-contact electrode values may increase uncertainties during force estimation. In this work, we make a hypothesis that the contact information can be estimated based on the contacted electrodes. Hence, the proposed force estimation method is implemented with the two steps: (1) the segmentation of the contact region, and (2) the computation of the contact information (i.e., contact force and contact location) based on the contacted electrodes. Figure 6 shows the computation process of the contact information.

To segment the contact region from the sensor surface, this work makes a hypothesis that the contact region contains the electrodes with a relatively high electrode value. This hypothesis is reasonable because the physical contact between the fingertip and the object increases the pressure in the contact region. Hence, the segmentation of the contact region is processed according to the two following steps: (1) the impedance in the tactile image $t_{img}$ is divided into several clusters; (2) the electrodes with a high proportional value are grouped to form the contact region Ω. In this work, we used GMM to cluster the tactile image. We use all pixel values in the tactile image $t_{img}$ to fit the GMM model denoted as Ω. The GMM is modeled as a mixture of *K* Gaussian distributions. The likelihood of an input *e* under a GMM is defined as

(5) $$P\left(e|t_{img}\right)=\sum_{k=1}^{K}\pi_{k}N\left(e|\mu_{k},\sigma_{k}\right),$$

where *K* is the number of the Gaussian components used in Ω. *K* is set to 3 in this work. The three clusters can represent three different contact states, i.e., contact, intermediate-contact, non-contact. *e* is one pixel value in the tactile image $t_{img}$. $\pi_{k}$ is the prior of the *k* Gaussian component. $N(x|\mu_{k},\sigma_{k})$ represents a cluster with the mean $\mu_{k}$ and covariance $\sigma_{k}$. The parameters $\left\{\pi_{k},\mu_{k},\delta_{k}\right\}$ are estimated by maximum likelihood on $t_{img}$.

After the GMM fitting, each cluster is described with its mean and covariance. The probability of a pixel belonging to each of the clusters can be calculated. In this work, we denote the cluster with highest electronic value at its cluster center as the contact region $P_{c}\left(e\right)=N\left(e|u_{c},\delta_{c}\right)$. A pixel *e* is said to contact the object, if the predicted probability $P_{c}\left(e\right)$ is more than a threshold α, as defined in Equation (6). In this work, the threshold α is set to 0.9. As a result, all pixels that are predicted as a contact are grouped to form the contact region denoted as $C_{img}$:

(6) $$f\left(e\right)=\left\{\begin{array}{cc} contact\; & p_{c}\left(e\right)>=\alpha ,\\ noncontact\; & p_{c}\left(e\right)<\alpha , \end{array}\right.$$

Given the segmented contact region $C_{img}$, we further compute the contact information (i.e., contact force and contact location). This work uses a Gaussian component to fit the $C_{img}$. The mean of the fitted Gaussian component is used as the contact location, which is denoted as $p=\{p_{x},p_{y}\}$. The contact force *f* is defined as a weighted sum of the values of the pixels that belong to $C_{img}$. Equation (7) shows the computation of the contact force. In this way, the influence of the non-contact electrodes is discarded during force estimation:

(7) $$f=\sum_{i}^{n}P_{c}\left(e_{i}\right)e_{i}$$

where *n* is the number of pixels in $C_{img}$. $P_{c}\left(e_{i}\right)$ is the predicted probability of the pixel *i* which reflects the degree of contact. $e_{i}$ is the value of the pixel *i*.

Finally, the contact information (i.e., $\Psi =\{f,p,C_{img}\}$) between a robotic fingertip and the object is computed from tactile data. The estimated contact information is further used as a reference signal for force feedback control.

### 3.3. Grasping Force Controller

A grasping force controller is employed to drive the robotic hand to keep a feasible grasping force to stabilize an object without damaging or dropping it. In this work, the grasping force controller is built based on force feedback control. The torque $\tau_{i}$ of each joint $q_{i}$ of the robotic hand is controlled individually to track the desired grasping force $f_{d}$ in Cartesian space. Hence, each finger of the robotic hand is controlled to maintain the desired grasping force to ensure the stability of the grasped object. In the work at hand, the desired grasping force $f_{d}$ is updated autonomously online according to the results of the slip and material detection. The following steps update the desired grasping force:

1. An initial grasping force $f_{0}$ is manually set for each finger of the robotic hand in the initial phase.
2. The initial grasping force $f_{0}$ is then updated to be $f_{d}^{m}$ according to the result of the material detection, as shown in Equation (8). The definition of the initial grasping force is related to the object material [8]. In the work, we use the detection material to update the initial grasping force. Usually, the object with foam material is usually lightweight and has a large friction coefficient. Thus, we use a smaller initial force for the object with foam material. The object with metal material has a relatively high weight and low friction coefficient. Thus, we use a bigger initial force for the object with metal material. Objects with different materials require different grasping forces to ensure the grasping stability:
  (8) $$f_{d}^{m}=\left\{\begin{array}{cc} f_{0} & c^{m}=c_{paper}^{m}\\ f_{0}-\delta f_{m} & c^{m}=c_{foam}^{m}\\ f_{0} & c^{m}=c_{plastic}^{m}\\ f_{0}+\delta f_{m} & c^{m}=c_{metal}^{m} \end{array}\right.$$
3. Once a slippage is detected during the grasping process, we increase the desired grasping force $f_{d}^{m}$ by adding a fixed amount of $\delta f_{s}$ resulting in $f_{d}^{s}$. In this work, the value of $\delta f_{s}$ is to 0.2. Otherwise, the desired grasping force $f_{d}^{s}$ is set to equal $f_{d}^{m}$, as shown in Equation (9):
  (9) $$f_{d}^{s}=\left\{\begin{array}{cc} f_{0} & c^{s}=c_{noncontact}^{s}\\ f_{d}^{m} & c^{s}=c_{contact}^{s}\\ f_{d}^{m}+\delta f_{s} & c^{s}=c_{slip}^{s} \end{array}\right.$$
4. The desired grasping force $f_{d}$ is finally obtained by clipping $f_{d}^{s}$ into a safety region with the maximum and minimum value (i.e., $f_{d,max}$ and $f_{d,min}$). During the grasping process, the desired grasping force $f_{d}$ is updated continually, the force feedback controller is employed to drive the robotic fingers to track $f_{d}$.

Given the updated desired grasping force $f_{d}$ as guidance, a force feedback controller is employed to drive the robotic fingers to track the $f_{d}$ to stabilize an object without damaging or dropping them. Figure 1 shows the diagram of the control architecture. The controller first takes the estimated contact force *f* as a feedback signal. The current contact force *f* is compared with the desired grasping force $f_{d}$ to calculate the force error Δf, i.e., $\Delta f=f_{d}-f$. This work use a Proportional-Integral-Derivative (PID) controller [34] for force feedback control, which takes the force error Δf as input and computes the target joint torque τ. The target torque is then sent to the low-level torque controller to control the robotic fingers. Algorithm 1 shows the process of object stabilization through tactile sensing.

**Algorithm 1**: Grasping force control through tactile sensing. **Requires**: a trained online detection module, tactile readings, a initial grasping force $f_{0}$ **Repeat**:  Generate a tactile sequence $t_{seq}$ and a tactile image $t_{img}$ from tactile readings  Perform online detection based on $t_{seq}$ and output contact event $c^{s}$ and object material $c^{m}$  Perform force estimation based on $t_{img}$ to calculate current contact information $\Psi =\{f,p,C_{img}\}$  **If**
$c_{s}=c_{non-contact}^{s}$
**then**   Perform position controller to control the joints of the robotic hand to reach desired joint position  **else**  Update the desired grasping force $f_{d}$ based on $c^{s}$ and $c^{m}$   Perform force feedback control to tracking the desired grasping force $f_{d}$


## 4. Experiments

In this section, we first evaluate the performance of the proposed tactile sensing method and the force estimation method. The effectiveness of the grasping force controller is then demonstrated in a real-world robotic experiment. The experiment results and their discussions are presented.

### 4.1. Evaluation of Online Detection Module

In this subsection, we first introduce the collected tactile dataset for slip and material detection. The performance of the online detection module is then evaluated over this dataset.

#### 4.1.1. Dataset and Implementation

A tactile dataset that is suited for the material and slip detection simultaneously is still missing in the robotic community. Hence, this work introduces a new tactile dataset that includes the tactile data, the ground-truth of material, and contact event. In this dataset, three contact events (i.e., *non-contact*, *contact*, and *slip*) and four different materials (i.e., *paper*, *foam*, *plastic*, and *metal*) are considered.

**Figure 7:**
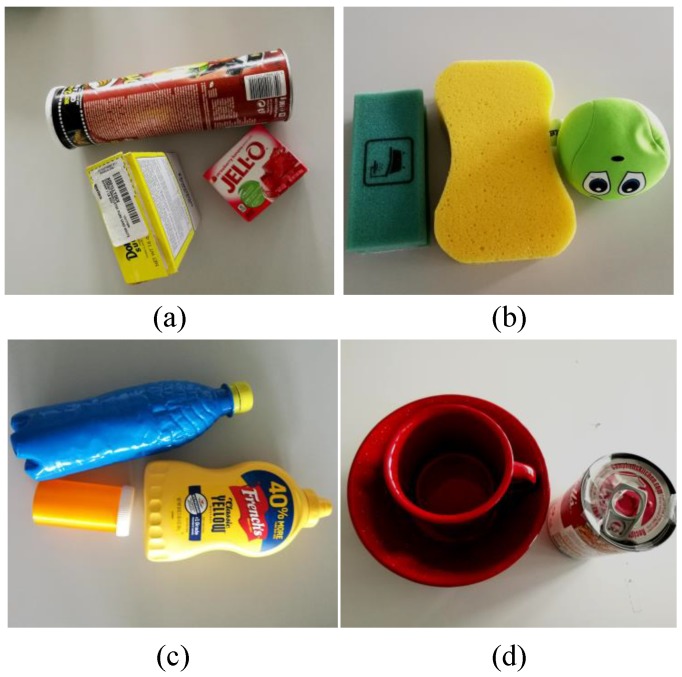
Twelve objects are contained in the tactile dataset. These objects were divided into four groups according to their different materials. (**a**) the *paper*, (**b**) the *foam*, (**c**) the *plastic*, and (**d**) the *metal*.

For tactile data collection, a Shadow Dexterous Hand equipped with BioTac sensors on its fingers is used. We use three fingers (i.e., thumb, first finger, and middle finger) of the Shadow Dexterous Hand for data collection. Twelve household objects were selected and divided into four groups according to their different materials, as shown in Figure 7. The data collection is performed to record the tactile data across all the objects under the three different contact events. We controlled these objects manually to interact (i.e., contact or slip) with the fingertip of the Shadow Dexterous Hand to produce tactile data. The tactile data were recorded at 100 Hz and saved into ROS bag files. The recorded tactile readings had 23-dimensional tactile features. As introduced in Section 3.1, the tactile sequence $t_{seq}$ with a dimension of l∗23 was extracted from the tactile readings and taken as samples for the dataset. The tactile sequences are sampled from these tactile readings. We use the estimated contact force for data segmentation. If the estimated contact force at each time-step exceeds 1.0, the tactile sequence is labelled as contact or slip, otherwise, the tactile sequence is labeled as non-contact. As a result, all tactile sequences $t_{seq}$ were labeled with the contact event (0: *non-contact*, 1: *contact*, 2: *slip*) and the material class (0: *paper*, 1: *foam*, 2: *plastic*, and 3: *metal*).

The training of the proposed online detection module is performed as follows: First, the proposed tactile dataset is used to train the LSTM-based encoder–decoder. The training of the encoder–decoder only requires the samples contained in the tactile dataset since the training process was in an unsupervised learning setting. The trained encoder–decoder is used to compute the latent represents *h* of all the samples. Next, we train the slip classifier and material classifier jointly by using all the latent representations of the samples and their corresponding labels. The training parameters of the online detection module are set as follows: The number of epochs is 20, the batch size is 5, and the learning rate is set as 0.00001. We use an Adam optimization method to optimize these two classifiers.

#### 4.1.2. Experimental Results

**Figure 8:**
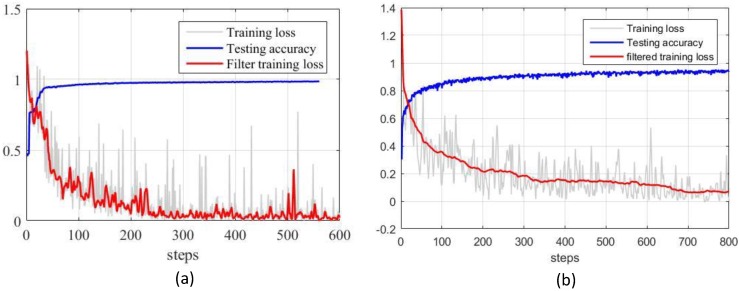
Performance of the proposed slip and material detection models. (**a**) Performance of slip detection, (**b**) performance of material detection.

The performance of the proposed online detection module is first evaluated based on the proposed tactile dataset. The dataset is split randomly into a training set (90%) and a testing set (10%). This work uses the training loss and the test accuracy for the evaluation. Figure 8 illustrates the training loss and testing accuracy of the slip and material detection. In terms of slip detection, the training loss is reduced as the training steps increase and converge to a minimum. The slip classifier achieves a high accuracy of about 98%. This means that the slip classifier can correctly classify the samples contained in the dataset. The high accuracy of the slip detection is of critical importance to the grasping force cotnrol since the accurate prediction of slippage guides the force feedback control. In terms of material detection, the detection accuracy is about 95%.

**Figure 9:**
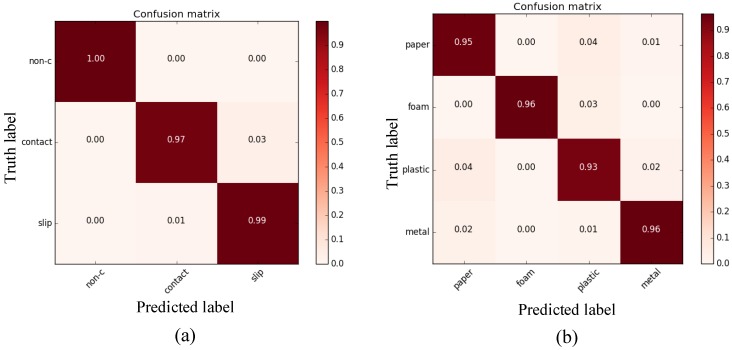
Two confusion matrices of slip detection (**a**) and material detection (**b**).

We then made use of two confusion matrices to evaluate the overall quality of the proposed detection module. Figure 9 shows these matrices which visualize the performance of the proposed slip and material classifiers. In each confusion matrix, each row shows the predicted probabilities for each ground-truth label. In terms of slip detection, the classifier could predict the contact event correctly since the diagonal elements have the highest values. There is no confusion between the slip and contact. In terms of material detection, the proposed material classifier is also able to predict the correct material for the object since the diagonal element has higher values than that of the other element in a small row. The *foam* material is classified with the highest accuracy. It is worth mentioning that several off-diagonal elements also have relatively high values. For example, the prediction results of the *plastic* material show a relatively high probability for the *paper* material. In this case, the proposed model may predict both object materials (i.e., *paper* and *plastic*) incorrectly. There is possibly confusion between the *plastic* and *paper* material because the two materials have similar physical properties, like the hardness.

Next, we analyzed the sensitivity of the proposed online detection module with respect to two important parameters (i.e., window size *l* and sample rate *f*). As introduced in Section 3.1, this work samples the tactile sequences $t_{seq}$ with a fixed window size *l* and sampling rate *f* from tactile readings for online detection. The window size *l* determines the number of sequential sensor readings that are taken as the input of the detection model. The sampling rate *f* determines how fast the sensor readings are taken. We evaluate how the performance of the detected module changes with respect to the two parameters. In this evaluation, three different window sizes l={16,32,48} and three different sampling rates f={50,33.3,25} Hz are selected. This means that the tactile sequence $t_{seq}$ is sampled every 2, 3, and 4 data points from the tactile reading. Before the sensitivity analysis, we first construct different tactile datasets by using the different combinations with the two parameters. The proposed detection module is then trained on these constructed datasets.

Table 1 shows the detection accuracy obtained from the comparison experiments concerning different parameters. The detection accuracy is used as the performance metric. First, it can be seen that the accuracy of the slip detection and material detection is improved by increasing the window size *l* with a fixed sampling rate. The highest accuracy is obtained with l=48. The reason for this behavior is probably that the bigger window size contains more tactile information that promotes the slip and material detection. Second, the detection accuracy does not improve with a higher sampling rate. When f=33.3 Hz, the detection performance is better than with the other two sampling rates. Finally, the highest slip detection accuracy of about 99% is obtained when the window size l=48 and sampling rate f=50 Hz or f=33.3 Hz. The highest material detection accuracy of about 97.2% is obtained, when l=32andf=25 Hz or l=48andf=33.3 Hz. Since the tactile data are sampled at 100 Hz in this work, the tactile sequence $t_{seq}$ needs 160 ms, 320 ms, and 480 ms of consecutive reading with window sizes of 16, 32, 48, respectively. As the window size increases, the detection delay is increased. To trade-off the real-time of detection and high accuracy, we chose a window size of l=32 and a sample rate of f=33.3 Hz for the following real-world experiments.

**Figure 10:**
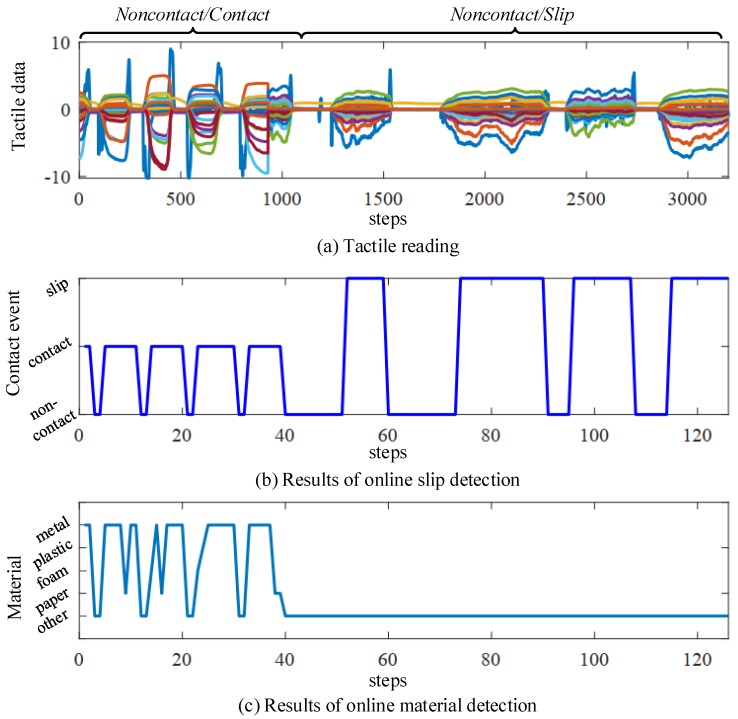
Examples of online slip detection and material detection using the tomato bottle. (**a**) the tactile reading is collected from the electrodes in the BioTac sensor. The first part of the tactile reading only consists of two contact events, i.e., *non-contact* and *contact*. The second part is *slip* or *non-contact*; (**b**,**c**) show the results of slip detection and material detection, respectively.

Finally, we show an example where the proposed detection module is used to detect contact events and object materials online. In this experiment, the tomato bottle is manipulated to interact with the thumb finger of the Shadow Dexterous Hand to produce tactile streaming. During the online detection process, the tactile sequence is continually sampled from the tactile readings and is sent to the proposed online detection module. The proposed detection module output the predicted results. Figure 10 shows the tactile readings and the detection results. First, Figure 10a shows the tactile readings recorded from the BioTac sensor. The truth label of the contact event is shown in Figure 10a. Figure 10b illustrates the results of slip detection. It can be seen that the proposed slip detection method can predict a correct contact event at each step. It is worth mentioning that there is a delay in the slip detection. The degree of delay is affected by the window size and sampling rate. Bigger window sizes and lower sampling rates would increase the delay of slip detection. Figure 10c shows the results of the material detection. In this work, we perform material detection only when the contact event is predicted as contact. Otherwise, the output of the material detection model is -1. Most times, the material of the object is predicted correctly. The proposed material classifier may predict the wrong material for the object due to sensor noise, as shown in Figure 10c. A good performance of online detection is of critical importance for the grasping force control. The proposed detection module is implemented in Python and runs on a 2.50 GHz Intel i5 CPU.

### 4.2. Evaluation of Force Estimation

**Figure 11:**
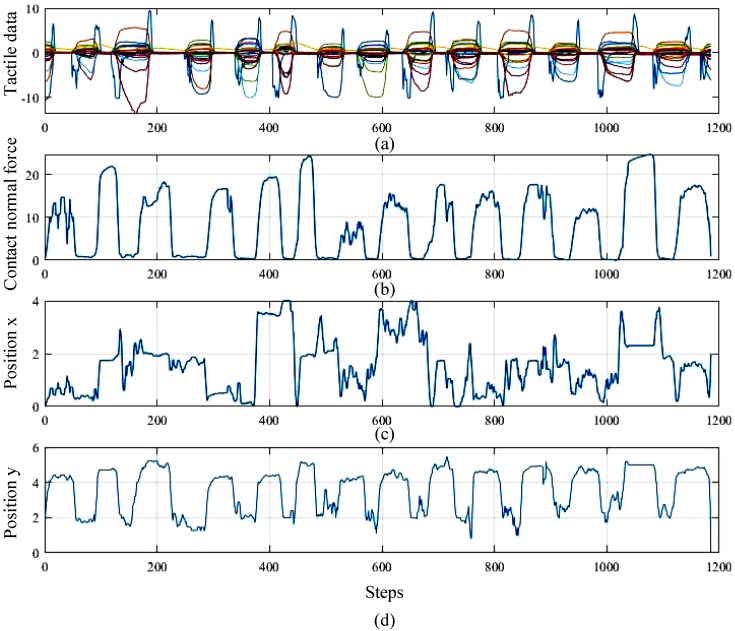
Illustration of force estimation. (**a**) the tactile data collected from the BioTac sensor; (**b**) the contact force; (**c**,**d**) show the contact location on the *x*- and *y*-axes.

The proposed force estimation method is used to compute the contact information (i.e., contact force and contact location) from tactile data. In this evaluation, we controlled an object manually to contact the fingertip of the shadow hand to produce tactile data. Figure 11 illustrates an example the force estimation. Figure 11a shows the tactile reading sampling from the electrodes of the BioTac sensor. In Figure 11b, it can be seen that the contact force changes when the robotic hand contacts the object. The contact location is also computed based on tactile data, as shown in Figure 11c,d. It is worth mentioning that the estimated contact force only describes the change of the contact state between the hand and the object. However, the estimated force is not a real force since no force calibration was performed. In future work, it is preferred to use a force/torque sensor for force calibration to obtain a real force value.

### 4.3. Real-World Grasping Force Control Experiment

**Figure 12:**
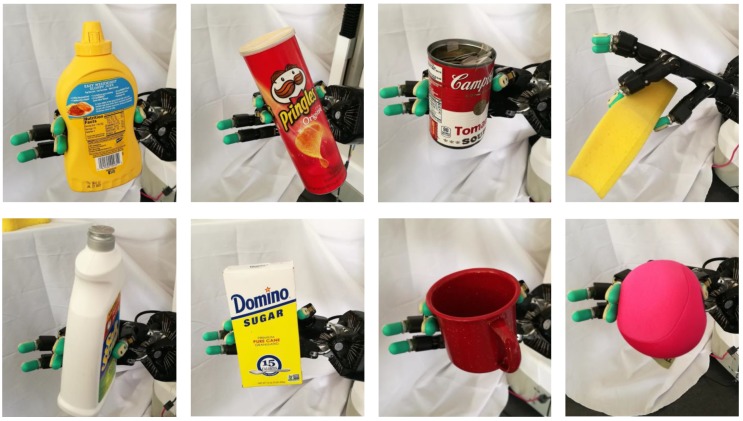
Object grasping with the Shadow hand using three fingers (i.e., thumb, first finger, and middle finger).

We use a Shadow Dexterous Hand with five fingers for this experiment. BioTac sensors are mounted on the fingertips of the Shadow Hand. Eight different objects are used, as shown in Figure 12. Each joint of the hand has its independent low-level torque controller. In this experiment, we considered grasp configuration involving three fingers (i.e., thumb, first finger, and middle finger) of the hand across all test objects. The BioTac sensors provide tactile data when the hand contacts an object. The goal of the grasping force control is to stabilize an object under physical uncertainties. In the grasping force control experiment, we adopt the following procedures: (1) The robotic hand in an open-hand configuration is controlled under position control to reach a specified position. (2) Once the online detection module detects contact between a finger and the object, the grasping force controllers are activated. (3) Humans exert physical disturbances on objects or the robotic hands manually. (4) The Shadow Hand adjusts its grasping force to stabilize the grasped object to advoid slippage. A video overview of the grasping force control can be found in the supplementary material.

**Figure 13:**
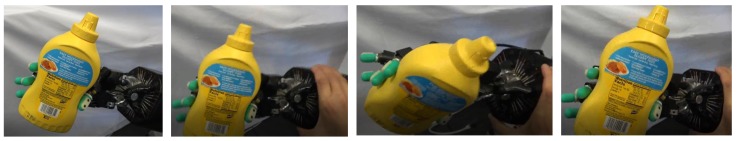
Illustration of the object grasping process under the external disturbance exerted by a user.

In a real-world application, a robotic hand may fail to grasp and hold an object by using a planned configuration due to the slippery surface and unknown weight of the object. Hence, we validate the proposed grasping force control strategy on a real robotic hand that implements object grasping tasks. The eight objects with different materials and weights were used. The Shadow Hand was controlled to grasp and hold an object. A user exerted a disturbance on the robotic hand manually by pushing and shaking it, as shown in Figure 13. A grasp is considered stable if the object was not dropped under the external disturbance. The grasp configuration with three contact points on the object surface is not enforced. We recorded ten trails for the eight objects. We use the success rate of the object grasping to evaluate the performance of the proposed framework. The final grasp success of object grasping is 95% (76/80). There were three failures when the robotic hand grasped the metal mug. One failure happened when the Shadow Hand grasped the chips can. Basically, the proposed strategy enables the Shadow Hand to stabilize the objects successfully. Figure 12 shows the results of grasping the objects with the Shadow Hand. By using the proposed control strategy, the contact force between the fingertip and the object is online adjusted to ensure grasp stability. The experimental results demonstrate that the proposed control strategies allow a multi-fingered robotic hand to stably grasp unknown objects without prior knowledge of their weight and physical properties.

### 4.4. Discussion

This work aims to explore a novel grasping force control strategy that combines tactile sensing techniques with feedback control to ensure a stable grasp with a multi-fingered robotic hand. Grasping force control of multi-fingered robotic hands is essential for a robot to implement manipulation tasks. However, it is still a challenge owing to the uncertainties arising from objects and environments. This work takes advantage of tactile information to address the problems of slip detection, material detection, and force estimation jointly. In this way, the uncertainties caused by the unknown object and the changing of environments are reduced during grasping force control of multi-fingered robotic hands. The robotic hand can update the desired grasping force automatically according to the results of tactile sensing. There have been some previous works that use tactile data to predict object materials [9] and contact events [5]. In contrast to these previous methods, we trained an online detection module to detect contact events and the object material simultaneously from tactile data. Moreover, the use of the low-dimensional latent representation of the tactile sequence in the detection model helps to reduce the model complexity. It enables different classifiers to share tactile features. In real-world robotic applications, it is of critical importance to build an online detection module instead of multiple separate detection models for sensory perception.

Grasp stability under external perturbations can be maintained by adjusting the grasping force [15] or by updating the contact point of the fingers on the object surface [17]. In this work, we consider adjusting the grasping force of a multi-fingered robotic hand to stabilize the grasped object. In the future, it will be beneficial to adjust the contact point and force of the fingers for a stable grasp. In this case, the global and local information of objects and the environment is required. Although tactile sensors provide critical information about interactions between the robotic hand and objects, tactile sensors only perceive local contact information. Visual sensors are one of the most mainstream sensors, which can provide global information about the target objects and the environment. It is interesting to fuse visual and tactile modalities for robotic perception.

In this work, the proposed method only identifies the material of the object surface. The classified material helps to define a rough initial grasping force for the robotic hand. However, an object may be made of several different materials. It is an very interesting research direction to do more refined material classification in the future. Meanwhile, object friction is also important information for robotic grasping and manipulation. If the friction of the object is detected, the choice of the initial grasping force can be more accurate. However, it is very difficult to build a training data set for friction classification. We cannot label the tactile data without professional measurements. In the future, we hope to build a training data set and extend the proposed online detection module to consider friction classification during object stabilization.

Finally, we discuss the generalization capability of the proposed methods. In this work, the proposed tactile sensing (i.e., slip detection, material detection, and force estimation) methods use tactile data produced by the BioTac sensor as inputs. Those methods are based on the BioTac sensor. Thus, the tactile sensing methods can not be transferred to other sensors directly. However, according to the characteristics of other sensor data, the proposed method can be applied by making changes accordingly. For example, in the force estimation method, the first calculation step, i.e., the generation of the tactile image, needs to be changed according to the inputted tactile data, and the subsequent calculation steps do not need to be changed. The grasping force controller can be transferred to other robotic hands.

## 5. Conclusions and Future Work

This work aims to enable robotic hands to make use of tactile sensing for the grasping force control. A grasping force control framework is proposed for a multi-fingered robotic hand to stabilize an unknown object online. By using this framework, multiple critical problems involving slip detection, object material detection, and force estimation are addressed simultaneously. We design an online detection module based on DNN to learn features automatically from tactile data for online detection. The detection module samples a tactile sequence online from the tactile readings and predicts the object material and contact event simultaneously. Meanwhile, a force estimation method exploiting the spatial property of tactile data is proposed to compute contact information (i.e., contact force and contact location). Hence, the spatio-temporal characteristics of tactile data are used for tactile sensing. By exploiting the results of tactile sensing, the grasping force control controller is employed to drive the robotic hand to adjust its grasp force to improve grasping stability. The effectiveness of the proposed framework was evaluated with a Shadow Dexterous Hand equipped with BioTac sensors.

Tactile sensors only perceive local contact information between the robotic hand and the grasped object. Visual sensors are one of the most mainstream sensors in the robotic community. They can provide information about the target object and the environment earlier. However, the effectiveness of visual perception is easily affected by lighting conditions or occlusion. Therefore, it is necessary to explore novel methods to fuse visual and tactile information for effective perception in the future. We also plan to perform the force calibration of the Biotac sensor based on a force/torque sensor to obtain ground truth force data.

## Figures and Tables

**Table 1:** Performance of the proposed detection module with respect to different parameters.

**(a) Slip Detection**			
	**Sampling Rate (** f **)**		
**Window size (** l **)**	**50**	**33.3**	**25**
16	96.9%	96.0%	97.6%
32	98.6%	98.6%	97.4%
48	99.0%	99.0%	98.0%
**(b) Material Detection**			
	**Sampling Rate (** f **)**		
**Window size (** l **)**	**50**	**33.3**	**25**
16	90.2%	90.7%	92.0%
32	94.5%	95.0%	97.2%
48	94.5%	97.2%	93.2%

## Supplementary Materials

The following are available online at https://www.mdpi.com/1424-8220/20/4/1050/s1, Video S1: Grasping force control with Shadow Dexterous Hand.
